# Total Face Approach (TFA) 3D Cephalometry and Superimposition in Orthognathic Surgery: Evaluation of the Vertical Dimensions in a Consecutive Series

**DOI:** 10.3390/mps4020036

**Published:** 2021-05-18

**Authors:** Giovanna Perrotti, Giulia Baccaglione, Tommaso Clauser, Riccardo Scaini, Roberta Grassi, Luca Testarelli, Rodolfo Reda, Tiziano Testori, Massimo Del Fabbro

**Affiliations:** 1 Lake Como Institute, 22100 Como, Italy; giovanna.perrotti@lakecomoinstitute.com; 2 Department of Orthodontics, University of Pavia, 22100 Como, Italy; giulia.baccaglione@gmail.com; 3 Department of Biomedical, Surgical and Dental Sciences, Università degli Studi di Milano, 20126 Milan, Italy; tommaso.clauser@unimi.it (T.C.); massimo.delfabbro@unimi.it (M.D.F.); 4 IRCCS Istituto Ortopedico Galeazzi, 20161 Milan, Italy; riccardoscaini@me.com (R.S.); tiziano.testori@unimi.it (T.T.); 5 Department of Biomedical Sciences, Sassari University, 07100 Sassari, Italy; grassi.roberta93@gmail.com; 6 Department of Oral and Maxillofacial Sciences, Sapienza University of Rome, 00161 Rome, Italy; rodolforeda17@gmail.com; 7 Department of Biomedical, Surgical and Dental Sciences, University of Milan, 20126 Milan, Italy; 8 Department of Periodontics and Oral Medicine, School of Dentistry, University of Michigan, Ann Arbor, MI 48109, USA

**Keywords:** total face approach, superimposition, orthognathic surgery

## Abstract

Background: Cephalometry is fundamental in diagnosis, analysis, and planning of orthodontic-surgical treatment as it reveals skeletal relationship between the upper and lower jaw as well as facial aesthetic parameters. Nevertheless, 3D cephalometry has still not become the exam of choice in orthognathic treatment even though today CBCT (Cone Beam Computed Tomography) is routinely used in other branches of dentistry. Methods: In a sample of 13 patients undergoing bimaxillary orthognathic surgery a chin-vertex CBCT exam was prescribed prior to orthodontic treatment (OT) and 12 months after surgery (T1). The DICOM files uploaded to MaterialiseSimplant Ortho software pro 2.1 (Materialise Co., Leuven, Belgium) were analyzed following the multiplane 3D Total Face cephalometry protocol (TFA). Results: Results comparing pre-op and post-op TFA 3D cephalometry, were then evaluated considering reference values reported in literature. The CBCT, carried out pre- and post-surgery, were subsequently analyzed employing the superimposition method using cranial base as reference. The aim of this study is to evaluate the advantages and disadvantages of the two methods in orthognathic surgery. Conclusions: Multiplane 3D TFA allows the clinician to locate where major or minor skeletal discrepancies are found with respect to ideal parameters and is also useful in classifying skeletal intermaxillary relation. The superimposition method is highly intuitive but does not provide information on the quantity and location of osteotomic movement.

## 1. Introduction

Orthognathic surgery is the branch of maxillo-facial surgery that corrects dentofacial deformity and related problems such as malocclusion and facial disharmony. By correctly repositioning the upper and lower jaws, functional, and aesthetic issues can be resolved. Candidates for this type of surgery present a three-dimensional malpositioning of the upper and/or lower jaw and a global facial analysis is necessary to treat this anomaly [1]. 

The current objective of orthognathic surgery is to establish a normal occlusion, i.e., class I molar and canine angulation with respect to Andrews’ six keys, overjet and overbite between 2 to 4 mm, coinciding median lines, and an adequate form and size of the arch. It is just as important that the occlusion allows the condyles to be correctly positioned inside the glenoid fossa, which is a fundamental prerequisite to have long-term functionality in the absence of TMJ disease. Other prerequisites include normal size respiratory pathways, and facial harmony with subsequent aesthetic and functional satisfaction of the patient with long-term stability of the result achieved [2]. 

In general orthodontics aims to achieve proper facial aesthetics with neuromuscular and occlusal balance. 

Two-dimensional (2D) cephalometry in orthodontics was introduced in early 20th century and over the years orthodontists used the exam evolving from hand-drawn sketches to software evaluation that allows computerized measurements [3,4]. Lateral cephalometric analysis obtained by 2D radiographs was for decades the method of choice to analyze the growth and development of cranio-facial structures to plan and evaluate orthodontic treatment. Nevertheless, obtaining 3D parameters of cranio-facial structures with this method had its limits, the parameters were modified or partially obscured leading to inaccuracies during the analysis [5]. 

A cephalometric image must be simple and essential, with clearly defined, and easily located points making measurements accurate and reproducible. The information obtained must be reliable providing skeletal and dental alveolar evaluation at various planes, and if the orthodontists and maxillofacial surgeons have a graphic representation, it allows intuitive and easy evaluation of the case. Although the use of CBCT has become routine practice in other branches of dentistry, 3D cephalometry has still not become the method of choice in orthognathic surgery.

Perrotti et al., devised a Total Face Approach (TFA) 3D cephalometric analysis system identifying new evaluation algorithms on vertical, sagittal planes linked with an evaluation module to determine the symmetry of the subject to create a new classification based on 3D data [6,7].

The CBCT, carried out pre- and post-surgery, can even be used employing the superimposition method taking the cranial base as reference. This study is the first study to present the use of TFA 3D cephalometry in a peer-reviewed international journal.

The aim of this study is to evaluate the vertical dimensions in patients who underwent orthognathic surgery utilizing 3D cephalometry vs. 3D CBCT models superimposition.

## 2. Materials and Methods

In this study a sample of 13 consecutive patients underwent bimaxillary orthognathic surgery. As per protocol, each patient had a chin-vertex CBCT exam before orthodontic treatment (OT) and again a year later (T1). Field of view (FOV) was large enough to incorporate all the maxillofacial area from the glabella to menton. The DICOM files utilized in our center come from one CBCT device (NewTom VGI, Verona, Italy) with a voxel size of 150 µm, with a mean absorbed dose of 100 µSv at full FOV of 15 cm. The DICOM files were uploaded to the MaterialiseSimplant O&O software (Materialise Co., Leuven, Belgium). The software was equipped with cephalometric analysis capability denoted Total Face Approach (TFA) and was employed to analyze all the cases enrolled in this study.

All radiographic exams where the head was not in the Natural Head Position (NHP) were excluded from the study. NHP was obtained by asking the patient to keep the head upright and relaxed while looking straight ahead into the reflection of his or her eyes in a mirror [8]. 

For each sample, a multiplane 3D cephalometry, defined TFA, was carried out for both of the CBCT exams prescribed. The cephalometric images regarding OT and T1 were compared in terms of cephalometric and post-op data following the TFA protocol [6]. The 3D cephalometry method used in this study is of the multiplane type. Three planes of reference are built: axial, sagittal, and coronal. They are independent from posture of the head with which the tomographic scan is acquired. Every point of these planes is identified on the 0.00 slice of its respective plane so that these remain external to the cranium. The analysis consists of a series of linear and angular measurements obtained by calculating the distance between the cephalometric points and planes built with reference to the three planes of the system. The vertical analysis method includes the calculation of the distance between the three planes of construction, passing through a cephalometric point and parallel to the Axial Plane. The construction planes are:

**SFP** (Superior Facial Plane): Plane passing through Nasion (N) point and parallel to Axial Plane.

**ANSP** (Anterior Nasal Spine Plane): Plane passes through the point corresponding to Anterior Spinal and Axial Plane.

**MP** (Mental Plane): Plane passing through the Menton (Me) point parallel to the Axial Plane.

The distances were always calculated between one point and one plane, in detail these are:

1. The Anterior-Superior Vertical dimension: distance between SFP and ANS point.
2. The Anterior-Superior Vertical dimension: distance between ANSP and Menton point.
3. Total Anterior Vertical dimension: distance between MP and the Nasion point.

Processing the data obtained from the three measurements of the vertical dimensions, intervals were created whose numerical values define the vertical properties of the subject under examination. The 3D classification involves the use of Perrotti et al. multiplane system [7]. The use of a colorimetric scale intended to simplify diagnostic data and make it more practical to use (Table 1).

The measurements were obtained automatically from the software and copied into an Excel table where the defects of the patient were determined before and after surgery allowing the quantification of the improvement of the defect.

Regarding the superimposition method, the 13 cases were inserted in the MaterialiseSimplant Ortho pro 2.1 (Materialise Co., Leuven, Belgium) software. At first automatic 3D rendering of the total volume was carried out, this was proceeded with introduction of a superimposition algorithm in the software. The procedure requires research and application of landmarks of reference that are not modified following orthognathic surgery: inferior orbital margins, zygomaticofrontal sutures, and upper and lower margins of the zygomatic arch.

The average range of skeletal displacement is also chosen, in this sample it was set at 15 mm. The software automatically carries out the pre- and post-op superimposition supplying a colorimetric visualization of the displacement.

## 3. Results

**Figure 1:**
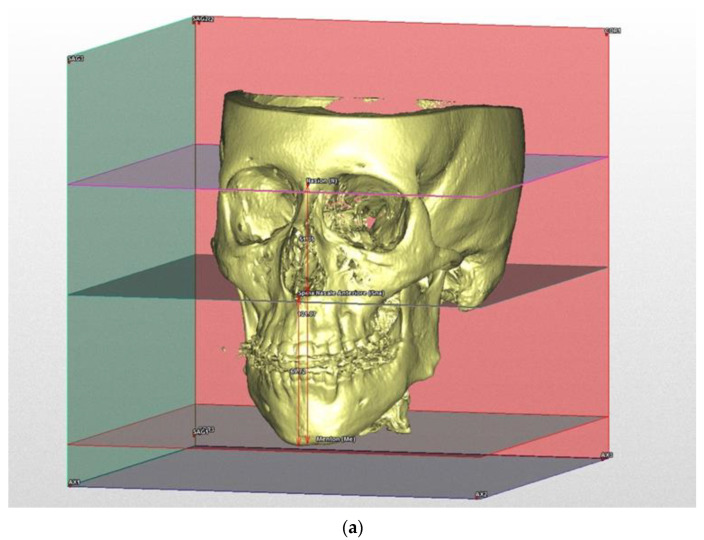

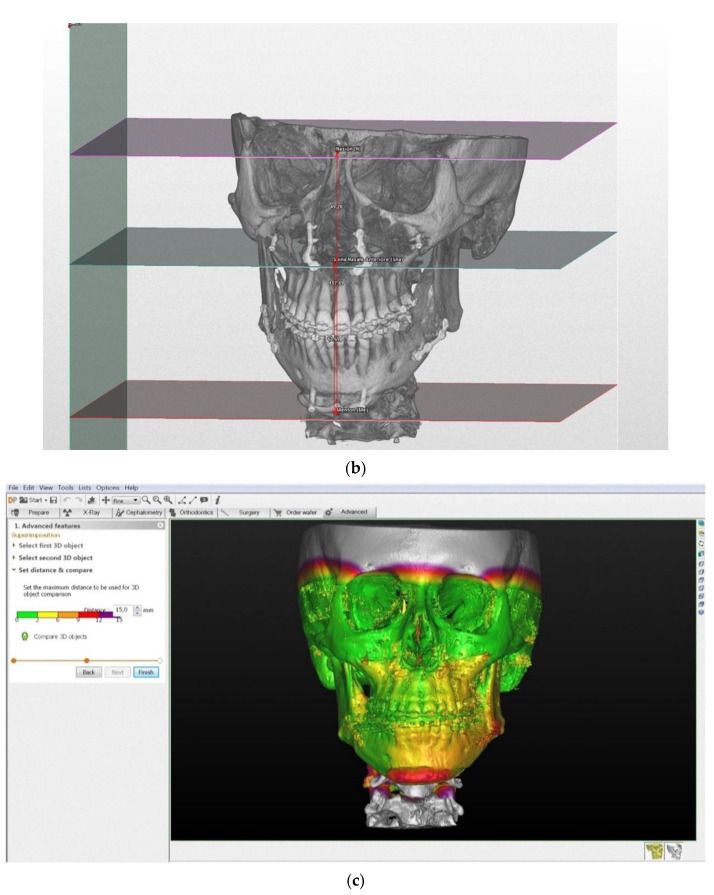
Clinical case of a patient with long-face biotype. Figures show TFA 3D multiplane cephalometry, before (**a**) and after (**b**) orthognathic surgery and superimposition (**c**).

**Figure 2:**
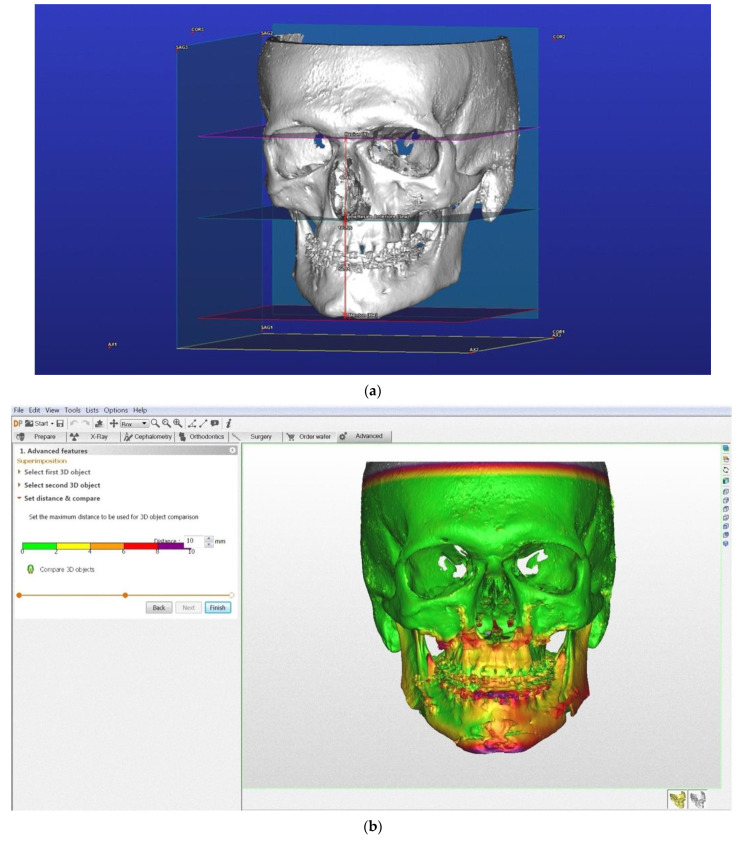

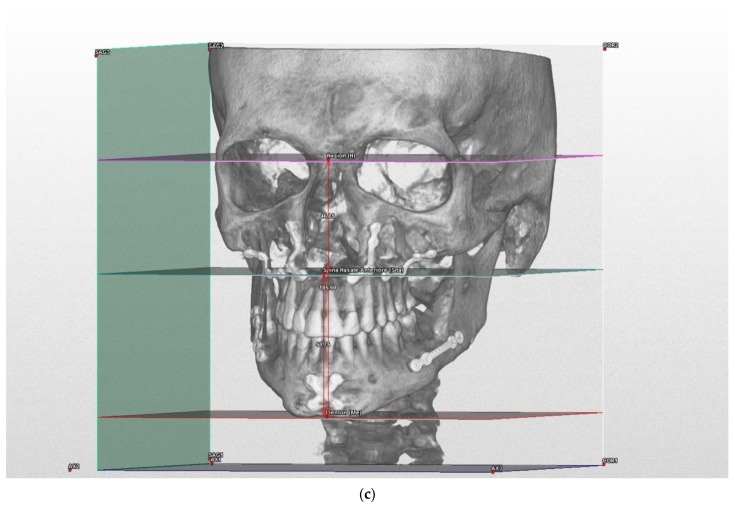
Clinical case of a patient with short-face biotype. Figures show TFA 3D multiplane cephalometry before (**a**) and after (**b**) orthognathic surgery and superimposition (**c**).

The results of pre- and post-op cephalometric analysis are reported in the tables (Table 2 and Table 3). A parameter of comparison between the TFA cephalometric method and superimposition was not found. Advantages and disadvantages of each method are however described in the table (Table 4 and Table 5). Figure 1 and Figure 2 show two clinical cases.

## 4. Discussion

TFA 3D cephalometry allows the clinician to locate where the major or minor skeletal discrepancies are located with respect to ideal parameters and is also useful in the classification of the patient which helps to determine whether there is a class I skeletal intermaxillary relation [9]. The superimposition method is highly intuitive but does not provide information on the quantity and location of osteotomic movement.

The purpose of 3D cephalometric analysis is to maintain the positive aspects of conventional 2D analysis but disposing of the negative features.

Three-dimensional cephalometric analysis is more complex compared to 2D analysis but provides reliable information regarding morphology, position, and orientation of anatomical structures via plane and specific point references.

Swennen et al. [10], published a study on 3D cephalometric analysis both for hard and soft tissues using linear, angular, orthogonal and proportional measurements between planes and points and points/planes but without indicating standard values.

Gateno et al. [4], described a new 3D cephalometric system analyzing the symmetry, transversality, verticality, pitch, sagittal dimension and form.

Farronato et al. [11], in a clinical study of 65 CT Cone-Beam patients with Ricketts class I and class II, with the help of dedicated software devised a cephalometric system based on 18 points that indicate the range of normality in patients.

Other studies [2,12,13] assessed the precision of the landmarks as these are considered the source of most errors in cephalometric analysis. This type of error is affected by many factors such as the quality of radiographic image, the reproducibility of landmark positioning, operator, and recording procedures.

Contrary to 3D cephalometry, when changes in the form and position of cranio-facial structures occur following surgery, the superimposition of volumes obtained by CBCT requires specific knowledge. Cevidanes et al. [14,15,16] were the first to introduce the method based on voxel for automatic 3D superimposition using the base of anterior cranium as the structure of reference. The quantitative changes are visualized via the colorimetric map which indicates the displacement of the three dimensions in space between various structures with respect to the cranium. This allows a visual evaluation of the position and extent of changes obtained with orthognathic surgery [17].

In this study 3D cephalometry was used, despite the limits inherent in cephalometry, it provided quantitative data that can be used as parameters to determine modifications that had really taken place. Three-dimensional cephalometry allows the identification of skeletal disharmony where greater or lesser discrepancies occur with respect to ideal values enabling the surgeon to target the intervention accurately. This method also allows a classification of the patient useful in determining whether skeletal and aesthetic correction were both achieved re-establishing a class I intermaxillary relationship. The advantages of TFA cephalometry are mostly due to speed of execution thanks to a user-friendly interface requiring a learning curve that is similar to traditional 2D cephalometry methods.

This 3D analysis was carried out evaluating the relationship between skeletal structures making the approach especially flexible and dynamic. The 3D visualization allows the determination of the extent of skeletal disharmony.

**Figure 3:**
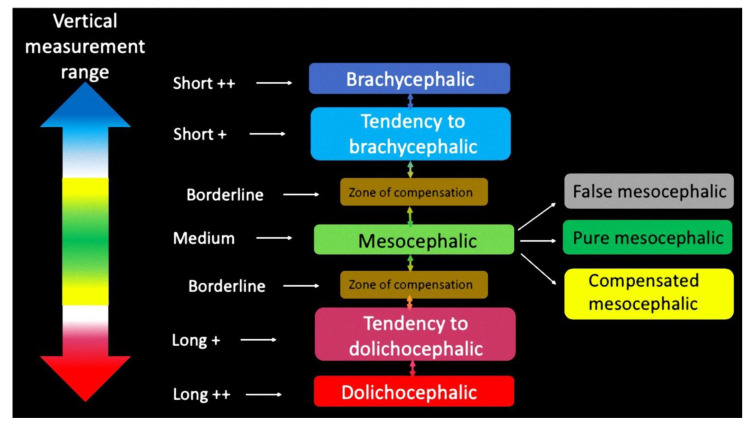
Diagnostic algorithm for the anterior-vertical measurement.

A diagnostic algorithm was created based on the summation of the upper vertical, lower and total dimensions. The result of this calculation shows the subject’s skeletal type analyzed from the point of view of the vertical dimension paying particular attention to the total vertical dimension (Figure 3).

The TFA method also has limits, interpreting TFA cephalometry detailed data are obtained but only on the skeletal base under consideration. Sometimes it is too wide to understand for instance whether the increase occurred at the pogonion or the entire mandibular base.

The 3D cephalometry starting from the axial, sagittal, and coronal images and the corresponding 3D renderings of the volumes allows the nosological subdivision of the patients into skeletal classes in accordance with similar models utilized in traditional 2D cephalometry; hence, the orthodontist and the maxillofacial surgeon can evaluate the surgical outcomes through objective data. Furthermore, the TFA 3D cephalometry allows the assessment of various bones not only singularly but also considering the right proportion with others. The 3D view allows the identification of a disharmonious relation among the skeletal bases where a lesser or greater discrepancy occurs in terms of verticality or sagitality.

Superimposition on the other hand is a method used in evaluating the orthognathic results whose range of evaluation is based on colorimetric parameters [14]. It is an intuitive method, but the colorimetric variations do not provide information on the extent of surgical correction achieved. The range of evaluation is also variable depending on operator. It would be necessary to reduce the range of colorimetry which would in turn make it more accurate and reduce interpretation errors. In our sample the variation range was set at 15 mm. It should be noted that in the case of the maxillary arches, variations can be much lower. Employing colorimetric analysis, it is also possible to evaluate the exact zone that underwent greater or lesser displacement. Unlike the TFA cephalometry method, superimposition can be carried out without any kind of software, and above all is fast necessitating the identification of only a few reference landmarks corresponding to points that underwent displacement via orthognathic surgery.

Superimposition analysis, however, does not allow patient classification, as in TFA method, which is useful to determine whether the patient has achieved skeletal, dental and aesthetic harmony.

Our study has some limitations due to the small sample size. Another limitation of the study is that the repeatability of the measurements could not be validated, since all the measurements were taken once by the principal investigator (GP).

## 5. Conclusions

Based on the available software ability, it was not possible to perform an objective comparative analysis between TFA and CBCT superimpositions.

TFA 3D cephalometry allows the determination of the extent of skeletal discrepancy with respect to ideal values and is also useful in the classification of the patient that helps verify whether class I intermaxillary relationship has been achieved.

The superimposition method aims to show the bone displacement on a colorimetric scale instead of a numeric one

However, our study has some limitations due to the small sample size. All the measurements were taken once by the principal investigator (GP). Intra- and inter-observers variability was therefore not measured.

## Figures and Tables

**Table 1:** Superimposition colorimetric scale.

					
0–3	3–6	6–9	9–12	12–15	mm

**Table 2:** Vertical Analysis of Patient Skeletal Class II.

Vertical Analysis of Patient Skeletal Class II															
	TFA									Superimposition (Highest Values)			Superimposition (Most Common Values)		
	Pre			Post			∆			∆			∆		
	N-Sna	Sna-me	N-Me	N-Sna	Sna-me	N-Me	∆ N-Sna	∆ Sna-Me	∆ N-Me	∆ N-Sna	∆ Sna-Me	∆ N-Me	∆ N-Sna	∆ Sna-Me	∆ N-Me
B.M.	51.3	69.72	121.07	48.4	68.04	116.44	2.9	1.68	4.63						
B.L.	46.72	54.75	101.47	45.3	60.63	105.93	1.42	−5.88	−4.46						
G.A.	51.74	66.62	118.36	46.61	70.26	116.86	5.13	−3.64	1.5						
H.A.	49.91	67.75	117.66	45.19	64.4	109.59	4.72	3.35	8.07						
S.L.	56.69	80.75	137.44	48.73	79.74	128.47	7.96	1.01	8.97						
S.D.	45.73	56.45	102.19	49.4	59.64	109.04	−3.67	−3.19	−6.85						
T.M	47.16	59.04	106.2	47.16	66.6	113.76	0	−7.56	−7.56						

**Table 3:** Vertical Analysis of Patient Skeletal Class III.

Vertical Analysis of Patient Skeletal Class III															
	TFA									Superimposition (Highest Values)			Superimposition (Most Common Values)		
	Pre			Post			∆			∆			∆		
	N-Sna	Sna-me	N-Me	N-Sna	Sna-me	N-Me	∆ N-Sna	∆ Sna-Me	∆ N-Me	∆ N-Sna	∆ Sna-Me	∆ N-Me	∆ N-Sna	∆ Sna-Me	∆ N-Me
B.F.	50.17	74.87	125.04	50.67	67.56	118.22	−0.5	7.31	6.82						
D.B.N.	52.78	73.7	126.48	53.24	73.85	127.09	−0.46	−0.15	−0.61						
M.S.	50.53	74.07	124.59	46.4	68.33	114.74	4.13	5.74	9.85						
P.E.	51.86	66.62	118.48	54.84	62.24	117.09	−2.98	4.38	1.39						
T.D.	53.02	73.06	126.07	55.28	72.88	128.16	−2.26	0.18	−2.09						
V.S.	51.09	73.46	124.55	48.86	65.14	114	2.23	8.32	10.55						

**Table 4:** Pros and Cons of TFA Multiplane 3D Cephalometry.

TFA Multiplane 3D Cephalometry	
Pros	Cons
Reference points available for patient classification	Does not indicate the skeletal base where the greatest variation has occurred
Allows extremely accurate evaluation	Limited software types available for this kind of analysis
An objective method	
User-friendly interface	
Fast execution	
Easy to learn	

**Table 5:** Pros and Cons of Superimposition.

Superimposition	
Pros	Cons
Highly intuitive, useful for the orthodontist, patient and maxillo-facial surgeon	Reference points not available for patient classification
Identifies the area with the greatest variation	Uses variable ranges that are operator dependent
Requires few reference landmarks in the areas not involved in the surgical variations	
Applicable to all available software	
Highly intuitive, useful for the orthodontist, patient and maxillo-facial surgeon	
Identifies the area with the greatest variation	
